# The Protective Effect of Salidroside on Hypoxia-Induced Corpus Cavernosum Smooth Muscle Cell Phenotypic Transformation

**DOI:** 10.1155/2017/3530281

**Published:** 2017-07-17

**Authors:** Xiang Zhang, Jian-feng Zhao, Fan Zhao, Jun-feng Yan, Fan Yang, Xiao-jun Huang, Gang Chen, Hui-ying Fu, Bo-dong Lv

**Affiliations:** ^1^The Second Clinical Medical College, Zhejiang Chinese Medical University, Hangzhou, China; ^2^Department of Urology, The Second Affiliated Hospital of Zhejiang Chinese Medical University, Hangzhou, China; ^3^Department of Urology, The Children's Hospital, Zhejiang University School of Medicine, Hangzhou, China; ^4^Andrology Laboratory on Integration of Chinese and Western Medicine, Zhejiang Provincial Key Laboratory of Traditional Chinese Medicine, Hangzhou, China

## Abstract

Salidroside, a major active ingredient isolated from* Rhodiola rosea*, has a long application in Chinese medical history. It has widely demonstrated effects on fatigue, psychological stress, and depression and exhibits potential antihypoxia activity. Emerging evidence shows that hypoxia is an important independent risk factor for erectile dysfunction (ED). The aim of this study was to clarify the effect of salidroside on hypoxia-induced phenotypic transformation of corpus cavernosum smooth muscle cells (CCSMCs). Our results showed that salidroside decreased the hypoxia-induced expression of collagen and content of vimentin, a corpus cavernosum smooth muscle synthetic protein, in vitro. Simultaneously, salidroside increased expression of the CCSMC contractile proteins, *α*-smooth muscle actin (*α*-SMA) and desmin. In vivo, similarly, the expressions of collagen and hypoxia-inducible factor-1*α* were increased in bilateral cavernous neurectomy (BCN) rats while they were decreased in the salidroside group. Among the phenotypic proteins, *α*-SMA and desmin increased and vimentin decreased after treating BCN rats with salidroside compared with the BCN alone group. Overall, our results demonstrate that salidroside has the ability to oppose hypoxia and can inhibit the CCSMC phenotypic transformation induced by hypoxia. Salidroside may provide a new treatment method for ED.

## 1. Introduction

Erectile dysfunction (ED) is considered a common clinical disease where the penis cannot reach or maintain sufficient erection to obtain satisfied coitus. Although ED is benign, it influences the body and mental health of patients. ED is closely related to the quality of life, sexual partner satisfaction, and stability of the patient's family. Phosphodiesterase type 5 inhibitors (PDE5-Is) are regarded as first-line treatment for ED [1]. PDE5-Is enhance erectile function by maintaining sufficient cellular levels of cyclic guanosine monophosphate (cGMP) in both the corpus cavernosum and its supply vessels, and the increased extension of the corporeal sinusoids allows greater blood flow. However, the total response rate for PDE5-Is in patients is poor; approximately 30–40% of men with ED do not respond to PDE5-Is therapy [2, 3]. These treatment failures may result from many different causes such as insufficient dose, insufficient sexual stimulation time, or even hypogonadism [4]. Following cavernous neurectomy, the release of NO in ED patients is partially limited because of cavernous nerve injury that affects the production of NO-induced cGMP. Therefore, the target pathway of PDE5-Is is disturbed under the condition of cavernous nerve injury [5] and it is important to identify a more effective drug and provide new clinical treatment methods.

In recent years, extensive research has shown that long-term hypoxia of cavernosum smooth muscle, which causes cell dysfunction, is one of the most important factors for ED [6, 7]. Hypoxia increases the expression of collagen in smooth muscle cells and, consequently, these cells fibrillate [8, 9]. As early as 1998, Moreland hypothesized that when the pressure of the erect penis sponge reached 90–100 mmHg, prostaglandin E increased, collagen was degraded, and differentiation of smooth muscle was maintained. When the pressure reached 25–45 mmHg in the soft penis, it promoted the expression of transforming growth factor-*β* [10]. Therefore, collagen was synthesized and connective tissue proliferated. Substantial research data show that many chronic hypoxia diseases, such as atherosclerosis, sleep apnea syndrome, and chronic obstructive pulmonary disease, can cause the disappearance of nighttime erections to differing degrees [11–13]. In contrast, the normal erection of a healthy man at night maintains the supply of oxygen and keeps the cavernosum smooth muscle contracted. This is an intrinsic physical mechanism to protect the normal form and function of cavernosum smooth muscle. However, hypoxia can cause phenotypic transformation resulting in the smooth muscle converting from a contractile to a synthetic state [14, 15]. Indeed, our previous study indicated that corpus cavernosum smooth muscle cells (CCSMCs) can shift from the contractile to synthetic phenotype under hypoxic conditions [16]. Therefore, antihypoxic therapy may be an effective means to treat ED.

Salidroside, a major active ingredient isolated from* Rhodiola rosea*, has been long used as a traditional Chinese medicine. Salidroside possesses various pharmacological properties and contributes to the physiological benefits of Rhodiola species that include resisting hypoxia, antiaging, anticancer, hepatoprotective, and neuroprotective effects [17–24]. In addition,* Rhodiola* species have been reported to promote longevity and work productivity [25]. In experimental studies, salidroside has markedly attenuated the hypoxia-induced loss of cardiomyocyte viability, necrosis, and apoptosis [26]. There is also evidence showing that salidroside can diminish cortical neuron cell death induced by hypoxia and decrease endothelial cell apoptosis by cobalt chloride-induced hypoxia [27]. Nevertheless, the ability of salidroside to inhibit hypoxia-induced changes on CCSMCs remains unclear.

The aim of this study was to reveal the ability of salidroside to resist hypoxia and inhibit phenotype modulation of CCSMCs. Clarification of the effect of salidroside on phenotypic modulation caused by hypoxia might provide a new therapy for ED and have significant clinical value.

## 2. Material and Methods

### 2.1. Reagents

**Figure 1:**
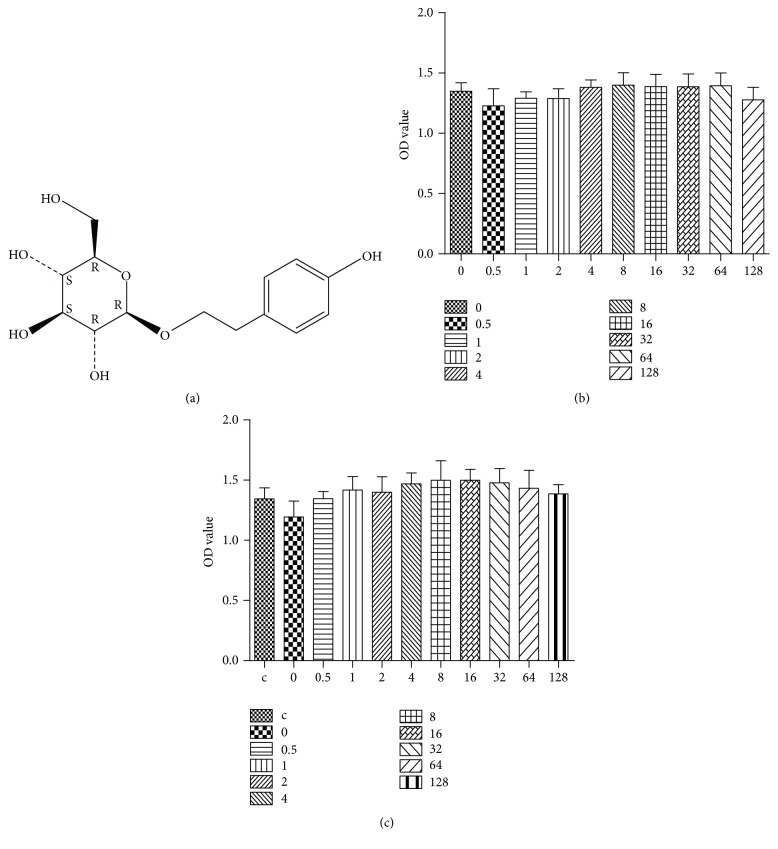
The effect of salidroside on cell viability. (a) The molecular structure of salidroside. Salidroside does not influence the viability of rat corpus cavernosum smooth muscle cells up to 128 *µ*g/ml using the MTT assay in normoxic (b) and hypoxic (c) environments. Data are expressed as means ± SE (*n* = 8; *P* > 0.05). c = normal oxygen group.

Salidroside (CAS registry number: 10338-51-9, batch number: Y18J8Y17024) was purchased from Yuan Ye Biotech. (Shanghai, China). According to the manufacturer's data, the purity was 98% based on HPLC analysis. The chemical structure is shown in Figure 1(a). 3-[4,5-Dimethylthiazol-2-yl]-2,5-diphenyl-tetrazolium bromide (MTT) was purchased from Sigma-Aldrich (St. Louis, MO, USA).

### 2.2. CCSMC Cultures and Treatments

CCSMCs were prepared from young male Sprague-Dawley rats (6 weeks of age) as described previously [28–30]. CCSMCs were incubated in 25 cm^2^ cell culture flasks. When the cell density reached 70–80%, the medium was removed and the cells washed twice with phosphate-buffered saline (PBS). Then, 1 ml pancreatin (Gibco, Grand Island, NY, USA) was added and the cells were incubated for 5 min. The mixed liquor was collected and centrifuged at 9600*g* for 10 min. The supernatant was removed and the pelleted cells were seeded in Petri dishes (100 mm diameter) in their growth medium. When the cells reached 70–80% confluence, the medium was replaced and cells were incubated in phenol red- and serum-free medium containing 0.1% bovine serum albumin (BSA, Amresco, Solon, OH, USA). After 24 h, salidroside was added at low (3 *µ*g/ml) and high (30 *µ*g/ml) concentrations, and the cells were placed in a hypoxic environment (1.5% O_2_, 5% CO_2_, and 93.5% N_2_) for 48 has described previously [31]. At the end of the experiment, cells were collected and further processed for protein extraction.

### 2.3. MTT Assay

Cells (7 × 10^4^/ml) were inoculated into 96-well plates. Each well contained 100 *µ*L. The medium was changed after complete adherence of the cells on the second day. Salidroside was then added at concentrations of 0, 0.5, 1, 2, 4, 8, 16, 32, 64, and 128 *µ*g/ml; every group contained 8 identical samples that were incubated for 48 h. Ten microliters of MTT (5 mg/ml) were added to each well. After 4 h, the supernatant was removed and replaced with 150 *µ*L dimethyl sulfoxide. The plates were oscillated for 10 min and the absorbance measured at 490 nm.

### 2.4. Animal Experiments

Adult male Sprague-Dawley rats (Shanghai Laboratory Animal Center, Shanghai, China) weighing 275–325 g were used. All experiments were conducted in accordance with the Zhejiang Chinese Medical University Guidelines for Animal Care and Use. The protocol was approved by the Committee on the Ethics of Animal Experiments of Zhejiang Chinese Medical University. Rats were separated into four groups of 12: sham-operated, bilateral cavernous neurectomy (BCN), BCN plus 6 mg/kg salidroside, and BCN plus 2 mg/kg tadalafil. Both drugs were dissolved in water and administered orally, daily for 12 weeks. Rats were housed under a 12 : 12 h light/dark cycle at 24 ± 1°C and had free access to food and water. All surgery was performed under sodium pentobarbital anesthesia, and all efforts were made to minimize suffering. Rats were anesthetized by an intraperitoneal overdose of sodium pentobarbital followed by cervical dislocation for euthanasia.

The BCN operation was conducted aseptically as described previously [32]. Rats were anesthetized with an intraperitoneal injection of sodium pentobarbital (30 mg/kg). An incision was made from the symphysis pubis to the mid-abdominal region, and the dorsum of the penis was exposed. The cavernous nerves were dissected and transected on both sides. For the sham-operated group, the cavernous nerves were dissected but not cut. The incision was closed with a two-layer interrupted suture. Antibiotics were fed orally to all rats for 3 days after the operation.

### 2.5. Measurement of Erectile Responses

Erectile responses were measured as described previously [30]. Three weeks after surgery, the apomorphine test was performed. Lights were first dimmed in a tranquil laboratory. Rats were then placed in a transparent observation chamber for about 10 min to adapt to the new surroundings. Apomorphine (0.05%) was dissolved in normal saline containing ascorbic acid. The apomorphine solution was injected (0.5 mg/kg) in the loose skin of the back of the neck. Erection of the penis was assessed over 30 min. An erection was only counted when the emergence of an engorged glans penis and distal shaft was observed.

### 2.6. Harvesting of Tissue

Twelve weeks following the BCN operation, rats were anesthetized with an intraperitoneal injection of sodium pentobarbital (30 mg/kg). The whole penis was harvested. Penile tissue was cut into two parts: one was paraffin embedded and used for structural analyses and the other stored at −80°C for western blot analyses of phenotypic protein markers. Histological analyses involved Masson's trichrome staining for collagen and immunohistochemical staining of phenotypic protein markers (*α*-smooth muscle actin (SMA), desmin, and vimentin) and hypoxia-inducible factor- (HIF-) 1*α*. All antibodies were from Abcam, Cambridge, UK.

### 2.7. Immunohistochemistry

To prepare tissues for immunohistochemistry, rat penis samples were removed and fixed rapidly in 4% neutral formalin. The tissue was then dehydrated and embedded in paraffin before being cut into 4 *µ*m sections. In brief, sections were deparaffinized and rehydrated and then subjected to heat-induced epitope retrieval. Endogenous peroxidases were inhibited with 3% hydrogen peroxide. Nonspecific antigens were blocked with 5% BSA. The slides were then incubated with the primary antibodies (*α*-SMA 1 : 100, HIF-1*α* 1 : 100, and vimentin 1 : 250) overnight at 4°C, rinsed 3 times in PBS for 5 min at room temperature, and incubated with a biotinylated secondary antibody (1 : 100). This was followed by incubation with the streptavidin-biotin peroxidase complex (1 : 100; Wuhan Boster Biological Technology, Wuhan, China). Immunohistochemical detection was performed with 3,3′-diaminobenzidine tetrahydrochloride following the manufacturer's instructions. Tissue sections were viewed with an Eclipse 80i microscope (Nikon, Tokyo, Japan) equipped with a camera. Images were captured using NIS-Elements 2.30 software (Nikon) at 40, 100, and 400x magnification. Five nonoverlapping images were captured from each slide at 400x. Some slides were stained with hematoxylin and eosin to observe the cellular morphology. Masson's trichrome staining was used to measure the extent of corpus cavernous fibrosis. Quantitative image analysis was performed with Image-Pro Plus 6.0 software (Media Cybernetics, Rockville, MD, USA) as described previously [33].

### 2.8. Sodium Dodecyl Sulfate Polyacrylamide Gel Electrophoresis and Western Blot Analyses

Penises, including the urethra and tunica albuginea, were removed. Tissue samples were lysed in lysis buffer (20 mM Tris [pH 7.5], 150 mM NaCl, 1 mM EDTA, 1 mM EGTA, 1% Triton X-100, 2.5 mM sodium pyrophosphate, 1 mM beta-glycerophosphate, 1 mM Na_3_VO_4_, and 1 *µ*g/ml leupeptin) on ice. After centrifugation at 12,000*g* for 10 min at 4°C, protein concentrations were determined using a bicinchoninic acid protein assay kit (Pierce, Rockford, IL, USA). Forty micrograms of protein were loaded onto 6–12% sodium dodecyl sulfate polyacrylamide gels, electrophoresed, and transferred to polyvinylidene fluoride membranes (Bio-Rad, Hercules, CA, USA). Samples were treated with 5% BSA to block nonspecific antigen interactions before the membranes were incubated with primary antibodies against HIF-1*α* (1 : 3000), *α*-SMA (1 : 5000), collagen-I, vimentin (1 : 5000), or desmin (1 : 500) overnight at 4°C. All antibodies were from Abcam except the desmin antibody, which was from Santa Cruz Biotechnology (Santa Cruz, CA, USA). After rinsing three times in PBS for 8 min at room temperature, the membranes were incubated with a horseradish peroxidase-conjugated secondary antibody.

### 2.9. Statistical Analyses

All experiments were performed in triplicate. Data were analyzed by the *t*-test when comparing between two groups and by one-way ANOVA when comparing between multiple groups using SPSS 16.0 software (IBM, Chicago, IL, USA). The LSD *t*-test was used to compare pairs if the ANOVA difference was statistically significant. Data are expressed as means ± SEM. *P* < 0.05 was considered to indicate statistical significance.

## 3. Results

### 3.1. Assessment of CCSMC Viability after Treatment with Salidroside

Different concentrations of salidroside were added into CCSMC cultures under normoxic and hypoxic environments. After 24 h, viability was assessed by the MTT method. Concentrations of salidroside up to 128 *µ*g/ml had no significant effect on viability in the normal oxygen and hypoxic environments (Figures 1(b) and 1(c)).

### 3.2. Collagen-I Expression

**Figure 2:**
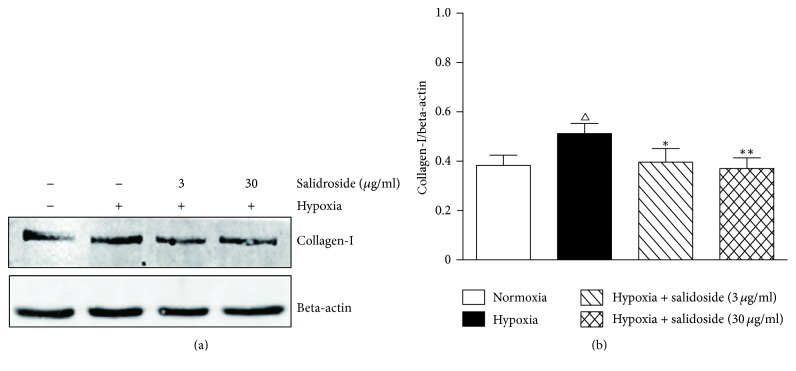
Salidroside prevents hypoxia-induced increases in the expression of collagen-I in rat corpus cavernosum smooth muscle cells. (a) A representative western blot of cell lysates from different treatment groups for collagen-I. Beta-actin was used as the loading control. (b) Quantification of the western blot data. Data are expressed as means ± SE. ^△^*P* < 0.05 compared to the normoxia group; ^*∗*^*P* < 0.05 and ^*∗∗*^*P* < 0.01 compared to the hypoxia group.

Western blotting showed that collagen-I expression was increased by hypoxia and decreased significantly by treatment with 3 or 30 *µ*g/ml salidroside (Figure 2).

### 3.3. Detection of Proteins Associated with the CCSMC Phenotype

**Figure 3:**
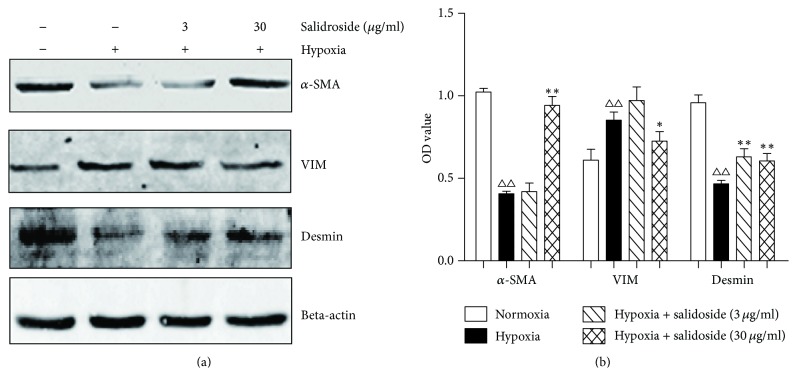
Effect of hypoxia on corpora cavernosum smooth muscle contractile proteins (desmin and *α*-smooth muscle actin (SMA) and a synthetic protein (vimentin)). (a) Representative western blots of desmin, vimentin, and *α*-SMA. Beta-actin was used as the loading control. (b) Quantification of the western blot data. Desmin and *α*-SMA are decreased and vimentin is increased in the hypoxic condition. Salidroside increases the expression of desmin and *α*-SMA and decreases the expression of vimentin in the hypoxic condition. Data are expressed as means ± SE. ^△△^*P* < 0.01 compared to the normoxia group; ^*∗∗*^*P* < 0.01 and ^*∗*^*P* < 0.05 compared to the hypoxia group.

Proteins related to smooth muscle contraction (*α*-SMA and desmin) were decreased in CCSMCs in the hypoxic environment compared with control cells. However, *α*-SMA and desmin levels increased after adding salidroside. The synthesis of a related protein, vimentin, increased after hypoxia and decreased in response to salidroside (Figure 3).

### 3.4. Detection of HIF-1*α* Expression In Vitro

**Figure 4:**
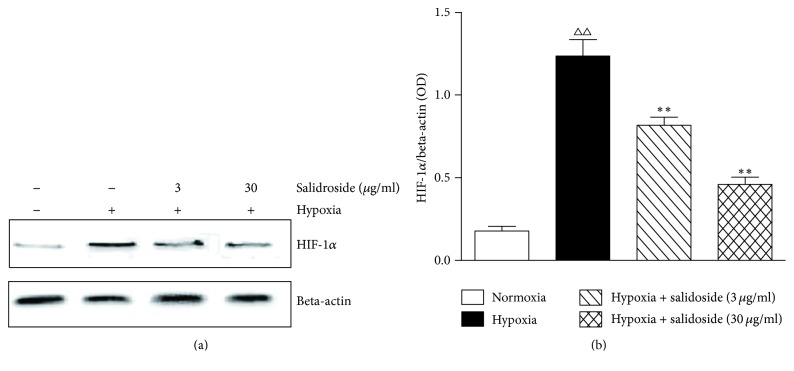
The expression of hypoxia-inducible factor- (HIF-) 1*α* in rat corpus cavernosum smooth muscle cells. (a) Representative western blot of HIF-1*α*. Beta-actin was used as the loading control. (b) Quantification of the western blot data. The hypoxic environment increases and salidroside decreases the expression of HIF-1*α*. Data are expressed as means ± SE. ^△△^*P* < 0.01 compared to the normoxia group; ^*∗∗*^*P* < 0.01 compared to the hypoxia group.

The expression of HIF-1*α* increased after exposure of CCSMCs to hypoxia. The low (3 *µ*g/ml) and high (30 *µ*g/ml) concentrations of salidroside decreased the expression of HIF-1*α* compared with the hypoxia group (Figure 4).

### 3.5. Assessment of Erectile Response

After injection with apomorphine, rats in the BCN group exhibited a lower erection rate than the sham-operation group (Table 1). No rats in the BCN group showed any erection within 30 min, while 11 rats showed a clear erection and one rat showed a slight erection in the sham group. Three rats in the salidroside group showed obvious erections while 9 rats showed no erection. In the tadalafil group, 4 rats erected and 8 rats did not erect.

### 3.6. HIF-1*α* Expression In Vivo

**Figure 5:**
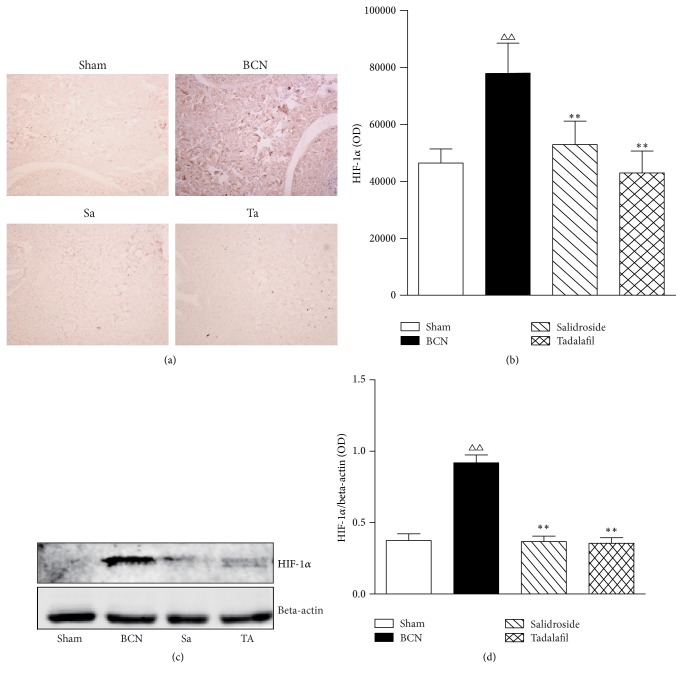
Salidroside decreases the expression of hypoxia-inducible factor (HIF)-1*α* in rat penile tissue. (a) HIF-1*α* was detected by immunohistochemistry. Photos were recorded using phase-contrast microscopy (magnification, ×400). (b) Optical density (OD) of the immunohistochemistry was determined using Image-Pro Plus 6.0 software. (c) Representative western blot of HIF-1*α*. Beta-actin was used as the loading control. (d) Quantification of the western blot data. Data are expressed as means ± SE. ^△△^*P* < 0.01 compared to the Sham rats. ^*∗∗*^*P* < 0.01 compared to the BCN rats (*n* = 12/group). Sham = sham-operated. BCN = bilateral cavernous neurectomy. Sa = BCN + salidroside. Ta = BCN + tadalafil.

Expression of the HIF-1*α* protein in penile tissue was evaluated by immunohistochemistry and western blot. Twelve weeks after surgery, very few areas in tissues from the sham group were positive for HIF-1*α* protein expression. In contrast, tissues from the BCN group exhibited strong HIF-1*α* expression in many areas (Figure 5(a)). The salidroside plus BCN and tadalafil plus BCN groups expressed a small amount of HIF-1*α* (Figure 5(a)). Quantitation of the immunohistochemistry (Figure 5(b)) and western blots (Figures 5(c) and 5(d)) confirmed these changes.

### 3.7. Assessment of Collagen Fiber Expression

**Figure 6:**
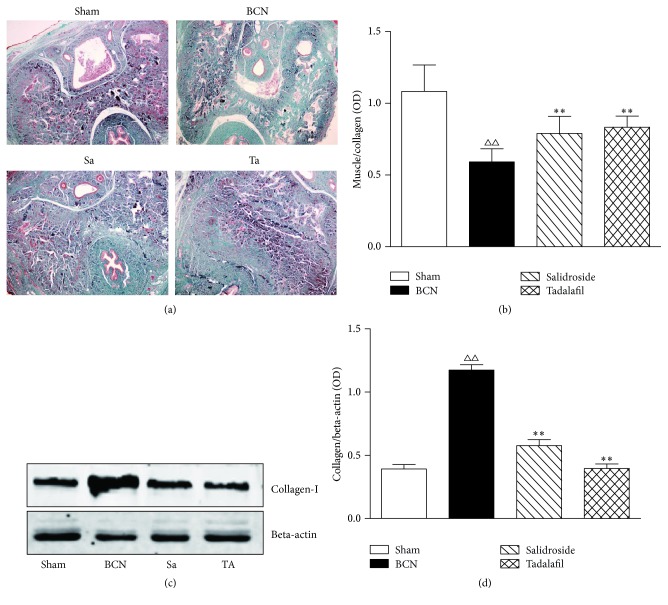
Salidroside decreases the expression of collagen-1 in BCN penile tissue. (a) Collagen and muscle were detected by Masson's trichrome staining. Photos were recorded using phase-contrast microscopy (magnification, ×400). (b) The muscle/collagen-1 ratio was detected by semiquantitative analysis of immunohistochemistry images of corpus cavernosum tissues. ((c), (d)) Collagen was detected by western blot. Beta-actin was used as the loading control. Data are expressed as means ± SE. ^△△^*P* < 0.01 compared to the Sham rats; ^*∗∗*^*P* < 0.01 compared to the BCN rats (*n* = 12/group). Sham = sham-operated. BCN = bilateral cavernous neurectomy. Sa = BCN + salidroside. Ta = BCN + tadalafil.

The extent of corpus cavernosum fibrosis was evaluated by Masson's trichrome staining for collagen (Figure 6(a)) and western blot (Figures 6(c) and 6(d)) of penile tissue for collagen-1. The degree of penile fibrosis in BCN rats was more severe than that in the sham group (Figure 6(a)). Collagen contents of the salidroside plus BCN and tadalafil plus BCN groups were decreased compared with the BCN alone group (Figures 6(a) and 6(c)). The muscle/collagen ratio was decreased significantly in BCN rats and was substantially, but not completely, rescued by salidroside or tadalafil treatment (Figure 6(b)).

### 3.8. Detection CCSMC Phenotypic Proteins

**Figure 7:**
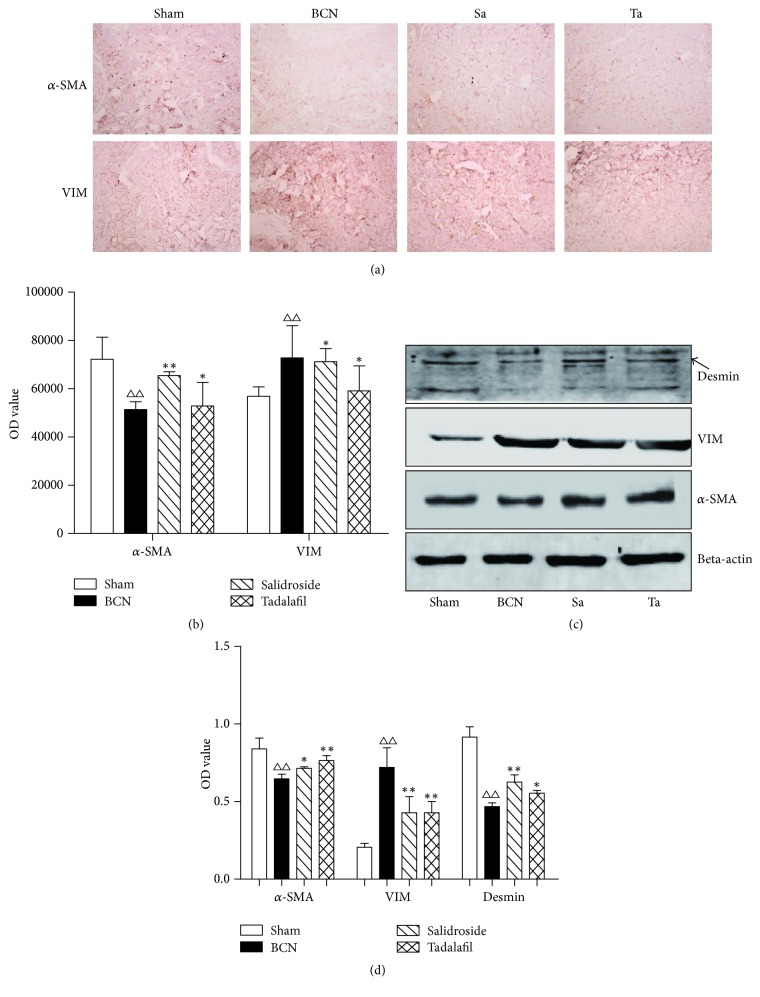
The effect of salidroside on the expression of corpora cavernosum proteins. (a) *α*-Smooth muscle actin (SMA) and vimentin were detected by immunohistochemistry. Photos were recorded using phase-contrast microscopy (magnification, ×400). (b) Semiquantitative image analysis of *α*-SMA and vimentin in different groups. (c) Representative western blot of proteins associated with the corpus cavernosum smooth muscle cell phenotype. Beta-actin was used as the loading control. Desmin signals are in the upper band. (d) Quantification of the western blot data. Salidroside increases the expression of the smooth muscle contractile proteins, desmin, and *α*-SMA and decreases the expression of the corpora cavernosum smooth muscle synthetic protein, vimentin. Data are expressed as means ± SE. ^△△^*P* < 0.01 compared to the Sham group; ^*∗∗*^*P* < 0.01 and ^*∗*^*P* < 0.05 compared to the BCN group (*n* = 12/group).

The expression of phenotypic marker proteins was evaluated by immunohistochemical staining and western blot of penile tissue (Figures 7(a) and 7(c)). Computer-assisted analysis of these images indicated that *α*-SMA expression decreased and vimentin expression increased significantly in the BCN rats compared with the sham group. After treatment with salidroside or tadalafil, *α*-SMA increased and vimentin decreased. Using western blot analyses, the expression of *α*-SMA and desmin was downregulated in the BCN compared with the sham group. In contrast, the expression of vimentin was upregulated. Salidroside or tadalafil treatment increased the expression of *α*-SMA and decreased the expression of vimentin. Semiquantitative image analysis of *α*-SMA and vimentin confirmed these results (Figure 7(b)).

## 4. Discussion

ED is a common clinical disorder with an increasing tendency to occur in younger individuals. It is predicted that globally there will be up to 322 million ED patients in 2025. Thus, ED is considered to be a significant health problem [34]. However the overall efficacy of PDE5-Is, the first-line drugs for ED, is only 60–70%. Basic research on ED indicates that hypoxia is one of the most important pathogenic factors. Salidroside, a traditional Chinese medicine extracted from* Rhodiola rosea*, has demonstrated antihypoxia and antiapoptosis activities. Accordingly, this study was designed to elucidate the ability of salidroside to prevent hypoxia-induced CCSMC phenotypic transformation.

According to our previous research and other reports [14–16], when hypoxia occurs in CCSMCs, *α*-SMA and desmin (contractile proteins of the CCSM) are downregulated, whereas vimentin (a smooth muscle synthetic marker protein) is significantly upregulated. The process that transforms CCSMCs from the contractile to synthetic state is known as phenotypic transformation. Our experimental results showed that salidroside increased the expression of *α*-SMA and desmin and decreased the content of vimentin. This was suggestive of an antihypoxic function of salidroside in CCSMCs.

HIF-1*α* is an important indicator of hypoxia. Its expression and activity are tightly controlled by the cellular oxygen concentration. Furthermore, HIF-1*α* plays an important role in the phenotypic transformation process in CCSMCs. Similar to our previous results [16], we showed that the expression of collagen in the extracellular matrix and HIF-1*α* levels increased significantly in CCSMCs in response to hypoxia. Collagen hyperplasia is a sign of fibrosis. In ED patients, collagen proliferates in the extracellular matrix. In penis tissue of ED patients, the cavernous smooth muscle ratio is decreased significantly (28–35%) compared with normal men (42–50%) [35]. Our experimental results showed that HIF-1*α* and collagen decreased after treatment with salidroside.

Tadalafil is a typical PDE5-I and induces erection by inhibiting the degradation of PDE5 and increasing the intracellular cGMP concentration. In our experiments, we chose tadalafil as the western medicine control to confirm the ability of salidroside to resist hypoxia and treat ED in BCN rats. Our results showed that salidroside, like tadalafil, could effectively prevent some effects of hypoxia related to ED. This was consistent with previous research. Salidroside decreased the expression of HIF-1*α* and retained the contractile properties of CCSMCs. At the same time, salidroside inhibited transformation of cavernous smooth muscle from the contractile to the synthetic state. This has important clinical significance to prevent the occurrence of ED induced by hypoxia.

However, the current study leaves some unanswered questions. First, it remains unclear whether once CCSMCs have transformed into a synthetic state in response to hypoxia, salidroside can reverse this change. Second, the exact molecular mechanism by which salidroside acts on CCSMCs is unknown. Finally, whether other models of ED, including the diabetic and vascular models, will yield the same result is not clear. Additional research is needed to answer these questions.

## 5. Conclusion

In brief, the results of our present study indicate that salidroside, a major active ingredient isolated from* Rhodiola rosea*, can resist hypoxia and prevent hypoxia-induced CCSMC phenotypic transformation through antihypoxia activities. Salidroside suppresses the switch of cavernous smooth muscle from the contractile to the synthetic state. Thus, it might have potential clinical value in preventing the occurrence of ED induced by hypoxia.

## Figures and Tables

**Table 1:** Penile erectile response to injection of apomorphine (0.5 mg/kg).

Group	Reaction	Nonreaction	Total	Erectile rate (%)
Sham	11	1	12	91.7
BCN	0	12	12	0^△△^
BCN + Sa	3	9	12	25
BCN + Ta	4	8	12	33.3

Data could not be analyzed by *R∗C* chi-square test because the samples were limited. Data were analyzed by Fisher's exact test. *α* = 0.05/3; ^△△^*P* < 0.01 compared to sham group. Sham = sham-operated. BCN = bilateral cavernous neurectomy. Sa = BCN + salidroside. Ta = BCN + tadalafil.
